# Case of Irregular Innervation

**Published:** 1870-12

**Authors:** Walter Hay

**Affiliations:** Chicago


ARTICLE VII. — *Case of Irregular Innervation.* By WALTER HAY, M.D., of Chicago.

The following analysis of a curious case of irregular innervation is presented in order to elicit the criticism of experts in nervous pathology, with the view to determine, if possible, its correct diagnosis, and suggestions for its treatment:

B—— ——, No. 80 Centre avenue, a robust Irish girl, has always enjoyed excellent health, aside from the nervous malady about to be described, which has existed from her earliest recollection, during which time the history of the case has been unmarked by any incident indicative of the cause of the malady, since its initial point, which appears to have been a severe fall upon the head sustained at the age of eleven months. The excellence of her general health is abundantly demonstrated by her present robust appearance, and the absence of any perceptible disturbance of the functions of organic life.

The characteristic features of the case are as follows:

1st, and most prominent (to her) partial *anæsthesia*, with *formication* of the surface and extremities, of the left arm, muscular power nearly normal, although her grasp of an object is not perfectly secure when her attention is diverted from it.

2nd. Constant *clonic convulsions* of the flexor muscles of the right arm, with *diminished muscular power* (*partial paralysis.*)

These conditions are occasionally apparent in the lower extremities in a lesser degree than in the upper, but generally their innervation is but slightly disturbed.

3rd. Pupil of the left eye nearly normal, somewhat contracted, perhaps, though the apparent contraction may be merely relative.

4th. Pupil of the right eye widely dilated and but slightly sensible to light.

5th. Visual range of the right (or dilated) eye about three times as long as that of the left (contracted eye), that is to say, objects just visible to the left eye at the distance of one foot and a half, are perceptible to the right eye at the distance of a little more than four feet.

6th. Reflex irritability of the spinal centres excessive, manifested in the right, but absent in the left or anæsthetic arm. Thus, if while carrying an object, such as some culinary utensil, dish, plate, etc., she should chance to be touched or spoken to suddenly and unexpectedly, the object would immediately be jerked upward by the convulsive movements of the flexors of the right arm.

7th. The point of the tongue when protruded is very slightly deflected to the right.

Having become habituated to this peculiar condition of the nervous system, the patient had regarded it as a subject for vexation or merriment rather than of anxiety, until recently, when its aggravation threatens to interfere with the performance of her daily avocation, that of a cook.

The diagnosis of such a case is of course purely inferential, to be demonstrated only by autopsy. The diminished (and altered) sensibility of the left arm would seem to indicate lesion, involving the posterior roots of the brachial plexus of the left side, together with more or less derangement of the gray substance of the central mass and of the posterior cornua of the opposite or right side above the 4th cervical vertebra, the plane of exit of the plexus from the cord, by reason of the decussation of the conductors of sensitive impressions in the gray substance of the cord. The gray substance, however, can be but partially involved, as from the almost perfect integrity of the innervation of the lower extremities, most of the conductors of sensitive impressions must remain intact.

The location of the lesion in this division and in this region of the spinal axis accounts likewise for the increased reflex activity in the muscles of the right arm, as the source of irritation must necessarily be above the corpora pyramidalia, the point of decussation of the voluntary motor conductors. Moreover, the partial structural integrity of the antero-lateral columns and of the anterior cornua of gray substance is demonstrated by the persistence (although diminished) of voluntary control of the muscular apparatus of the affected member.

This great reflex activity, together with the absence of hyperæsthesia, demonstrate the integrity of the posterior columns of the cord. The next point to be considered is the divergence of the tongue to the right, for the causes of which we must look back to the origin of the hypoglossal nerve in the groove between the corpora pyramidalia and olivaria (Gray), or from the gray nucleus upon the floor of the medulla oblongata (4th ventricle ?) (Stilling), near the posterior median furrow. In the same region of the cord, likewise, we find the origin of the two roots of the fifth nerve to which we must look for the sources of the perverted innervation of the iris of the right eye. The apparent difference in sensibility of the two retinæ is probably rather apparent than real, the greater range of vision in the right eye being due rather to the larger number of visual rays permitted to impinge upon the retina by reason of the greater degree of dilatation of the pupil of that side.

The absence of paralysis forbids the supposition of any structural change in the conductors of volitional impressions. The morbid phenomena seem to classify themselves under the two heads, *i. e.* diminution of sensitive, increase of motor energy; from which the inference seems logical, that the former is centric, originating in structural lesion of the gray substance of the cord, while the latter is a reflex effect of (probably) the same cause.

